# Disruptions to naloxone training among lay and occupational responders in Maryland during the emergence of COVID-19: Early impacts, recovery, and lessons learned^☆^

**DOI:** 10.1016/j.dadr.2023.100173

**Published:** 2023-06-16

**Authors:** Himani Byregowda, Catherine Tomko, Kristin E. Schneider, Erin Russell, Renee M. Johnson, Ryoko Susukida, Saba Rouhani, Taylor Parnham, Ju Nyeong Park

**Affiliations:** a Department of Mental Health, Johns Hopkins Bloomberg School of Public Health; b Department of Health, Behavior and Society, Johns Hopkins Bloomberg School of Public Health; c Center for Harm Reduction Services, Maryland Department of Health; d Department of Epidemiology, New York University School of Global Public Health; e Division of General Internal Medicine, Warren Alpert Medical School, Brown University

**Keywords:** Naloxone, COVID-19, Harm reduction, Overdose prevention

## Abstract

**Background:**

Opioid overdose death rates increased during the COVID-19 pandemic. Disruptions in community-based naloxone trainings could have reduced the likelihood of overdose reversal and increased the chances of a fatal overdose. We investigated changes in the number of people trained in naloxone administration and distribution in Maryland before, during, and after COVID-related stay-at-home orders.

**Methods:**

Data on naloxone training are from the Maryland Department of Health. We used interrupted time series models to estimate changes in average monthly number of people trained: [1] pre-interruption (4/2019–3/2020), [2] 1-month post-interruption (4/2020–5/2020), and [3] 12-month post-interruption (4/2020–3/2021). Trainees were classified as lay (e.g., people who use drugs) or occupational (e.g., law enforcement officers and harm reduction workers) responders.

**Results:**

There were 101,332 trainees; 54.1% lay, 21.5% occupational, and 23.4% unknown responder status. We observed a decrease in the average monthly number of trainees in the pre-interruption period (-235, *p*<0.001), a larger decrease of 93.2% during the 1-month post-interruption (-846, *p* = 0.013), and an increase during the 12-month post-interruption (+217, *p*<0.001). There was a significant decrease among occupational responders 1-month post-interruption, and a significant increase among lay responders in the 12-month post-interruption period.

**Conclusions:**

Findings suggest a marked decrease in naloxone trainees immediately after stay-at-home order, followed by a moderate rebound in the 12-months after stay-at-home order. The decrease in occupational responders trained may have limited access to naloxone, but would likely have been offset by increases in number of lay responders trained. Strengthening lay and occupational responder connections could maintain naloxone distribution during public health crises.

## Highlights

• Immediate drop in occupational naloxone trainees after COVID-19 stay-at-home order.
• No change in lay naloxone trainees immediately after COVID-19 stay-at-home order.
• Naloxone trainings recovered over the 12-month period after stay-at-home order.
• Strengthening connections between responder types can help maintain naloxone access.
• Programs should consider alternate naloxone delivery models for future emergencies.

## 1 Introduction

Opioid overdose continues to be a major public health problem globally, and in the US (World Health Organization, 2021), and there are indications that the COVID-19 pandemic or its associated consequences (e.g., closures of businesses and workplaces) exacerbated the problem (Centers for Disease Control and Prevention, n.d.). The US drug overdose death rate increased by 31% from 2019 to 2020 (i.e., 21.6 to 28.3 per 100,000 persons), the year the pandemic emerged (Hedegaard et al., 2021). The mean emergency department (ED) visit rate for opioid overdose in the US was significantly higher in March-October of 2020 compared to the same time period in 2019 (i.e., 336.7 per 100,000 vs. 220.4 per 100,000) (Holland et al., 2021).

Explanations for the increase in overdose during the emergence of the COVID-19 pandemic include solitary drug use during isolation, decreased access to substance use disorder (SUD) prevention and treatment services, changes in the composition of illicit opioids and patterns of use, and increased social and economic stress (Faust et al., 2021; Friedman and Akre, 2021). An additional explanation is that there were disruptions in harm reduction programs that distribute naloxone and offer instruction in its use. A decline in naloxone training programs could have reduced the likelihood of overdose reversals and increased the chances of a fatal overdose. Several studies are investigating how COVID-19 impacted overdose, so as to generate information about how to mitigate increases in overdose during public health emergencies. In the present study, we examine changes in the number of people trained by health officials to distribute and administer naloxone before, during, and after the implementation of the COVID-19-related statewide stay-at-home order in the state of Maryland.

### 1.1 Community-Based naloxone training and distribution

Naloxone is an opioid antagonist that rapidly reverses the effects of an opioid overdose. Distribution of naloxone and training on how to use it has been cited by CDC and the World Health Organization as a key strategy for reducing opioid overdose fatalities (Centers for Disease Control and Prevention, 2022a; World Health Organization, 2021; Doe-Simkins et al., 2014; Walley et al., 2013). To ensure use of naloxone to prevent overdose deaths, community programs prioritize training and distribution to people who are likely to be in a position to reverse an overdose. This includes people who use drugs (PWUD), their family and friends, and people who come into contact with PWUD through their occupation.

Typically, people working in community-based distribution of naloxone receive training from state or local agencies. As part of their training, they receive: [1] education about what naloxone is and how to administer it, and [2] naloxone kits to distribute. A brief naloxone training session that educates community members and health and safety workers on overdose identification and response is sufficient to improve comfort and competence in recognizing and administering naloxone in the event of an overdose (Behar et al., 2015).

Several countries permit naloxone to be sold over-the-counter (World Health Organization, 2021), and recently, the naloxone nasal spray was approved for over-the-counter use without a prescription in the US (Food and Drug Administration, 2023). However, naloxone was regulated as a prescription medication in the US during and after the emergence of the COVID-19 pandemic, and community programs were the main source of naloxone. To facilitate community distribution of naloxone, states and cities have “standing orders”, which enable community programs to distribute naloxone in compliance with state regulations with oversight from state agencies.

### 1.2 Pandemic-related changes in community-based naloxone training and distribution

The stay-at-home orders and social distancing protocols that were implemented as part of the public health response to COVID-19 pandemic may have impacted community-based naloxone programs (Courtemanche et al., 2020). In the US, the COVID-19 pandemic emerged in March 2020, and there were widespread closures of schools, clinics, and workplaces by the beginning of April 2020 (Raifman et al., 2020; Skinner et al., 2022). Community programs and harm reduction organizations were forced to limit operating hours and faced challenges in naloxone distribution efforts (Glick et al., 2020).

Decreases in naloxone training programs during the early days of the pandemic would likely have resulted in a decrease in access to naloxone in the community. However, there are indications that organizations pivoted operating procedures and revised outreach efforts to ensure service provision despite stay-at-home orders and social distancing protocols (Collins et al., 2020). Program adaptations included telephone-based opioid overdose education, online naloxone trainings, drive-through services, and expansion of mail-based naloxone distribution (Barnett et al., 2021; Courser and Raffle, 2021; French et al., 2021; Hughes et al., 2022; Krawczyk et al., 2021). It remains unclear whether there was sufficient access to naloxone during the emergence of the COVID-19 pandemic.

### 1.3 Current study

We investigate whether there were disruptions in community-based naloxone training in the state of Maryland after the emergence of the COVID-19 pandemic. Maryland is among the US states that have been highly affected by the opioid overdose crisis, due in part to the widespread proliferation of illicitly manufactured fentanyl (IMF) in the state's illicit opioid drug market. Maryland's opioid overdose death rate rose steeply during COVID-19, from 34.0 deaths per 100,000 in 2019 to 40.4 deaths per 100,000 in 2020 (Centers for Disease Control and Prevention, 2022c; 2023).

Maryland has a robust statewide program for community-based naloxone distribution led by the Center for Harm Reduction Services (CHRS), within the Maryland Department of Health (MDH). CHRS authorizes individuals and local entities to dispense naloxone in community settings, including local health departments, nonprofit organizations, law enforcement agencies, SUD treatment programs, and syringe service programs. Dispensing outside of the patient-provider relationship is permitted through a state standing order (Maryland Code Health, n.d.). CHRS provides training on naloxone administration and distributes naloxone to local entities free of charge to ensure broader access among people who are likely to witness or experience an overdose. CHRS took steps to maintain operations during the COVID-19 pandemic, although the extent to which training and distribution was sustained is not known.

The purpose of this study was to examine whether there was a decrease in naloxone training after the emergence of the COVID-19 pandemic. We compare the number of people who received training in naloxone administration and distribution from CHRS before, during, and after the implementation of Maryland's stay-at-home order, which was issued in March 2020. We specifically investigate changes based on different types of trainees, i.e., lay responders (e.g., PWUD and their close contacts) and occupational responders (harm reduction workers, community volunteers, and law enforcement officials).

## 2 Material and methods

### 2.1 Data on naloxone training and distribution

We used CHRS program data on naloxone training and distribution from April 2019 to March 2021. CHRS monitors and coordinates community naloxone distribution efforts and provides technical assistance and guidance to local entities engaged in distribution, i.e., Overdose Response Programs (ORPs). ORPs are required by the state of Maryland to submit monthly reports of naloxone training events to CHRS through a web-based form. The data represent naloxone training and distribution by ORPs and does not reflect the total amount of naloxone dispensed in the entire state by other sources, such as pharmacies. Because CHRS does not collect any identifiable personal information, individuals in the dataset are not necessarily unique individuals, as someone may receive multiple trainings and doses of naloxone from a single ORP over a given time period. Analysis of program data was exempt from review by the Johns Hopkins Bloomberg School of Public Health Institutional Review Board.

### 2.2 Study variables

We calculated the monthly number of people trained in overdose response education and naloxone administration by ORPs from April 2019 through March 2021. The date of naloxone training or distribution events was used to derive the number of people trained per month. Although doses of naloxone distribution are a more common measure in the literature, examining the number of people trained allows the estimation of the capacity needed at the state-level to conduct naloxone trainings.

The study period was divided into pre- and post-interruption periods based on the date of issuance of Maryland's stay-at-home order, with “interruption” referring to implementation of the order. The statewide stay-at-home order was issued on March 30, 2020 and lasted until May 15, 2020 (*State of Maryland*, 2020). The interruption period was April 2020, which included the only full month of the stay-at-home order. The pre-interruption period (April 2019-March 2020) represented the 12 months prior to the implementation of the stay-at-home order. We derived a 12-month post-interruption period (April 2020-March 2021). Within the post-interruption period, we also derived a 1-month post-interruption period (April 2020-May 2020) to examine immediate changes.

Trainees were classified as: (1) lay responders including PWUD, family and friends of PWUD, and others interested in receiving training; (2) occupational responders including law enforcement officials, harm reduction workers, community volunteers, and people who interact with PWUD in an occupational capacity, or (3) unknown. The occupational responder group does not include EMS personnel, who receive naloxone and training in its use through their organizations. The total sample included lay and occupational responders, as well as trainees with unknown responder status.

### 2.3 Statistical analysis

Descriptive statistics were used to summarize the total number of people trained per month, number of people trained by responder group, and doses of naloxone distributed. Next, Interrupted Time Series (ITS) analysis was conducted to examine whether the implementation of COVID-19 related stay-at-home order was associated with a change in the number of naloxone trainees. We chose this approach because the pandemic-related stay-at-home order was well-defined and implemented statewide, and because data on the number of naloxone trainees before and after the stay-at-home order was available. We estimated the average monthly number of trainees during the pre-interruption period (April 2019 to March 2020), the 12-month post-interruption period (April 2020 to March 2021), and the 1-month post-interruption period (April 2020 to May 2020). We used the ITS model to assess the statistical significance (*p*<0.05) of change in the average monthly number of trainees: [1] during the 12-month post-interruption period relative to the pre-interruption period, and [2] during the 1-month post-interruption period relative to the pre-interruption period. Separate ITS models were conducted for occupational responders, lay responders, and for the full sample of trainees.

To account for monthly fluctuations, a smoothing technique was used to average estimates of the monthly number of trainees. Newey-West standard errors were used to adjust for heteroskedasticity and autocorrelation (Linden, 2015), and the Cumby–Huizinga test was used to assess autocorrelation and include an appropriate lag term (Cumby and Huizinga, 1992). Coefficients represent the average change per unit (i.e., month) for the time-period; negative numbers indicate an average decrease and positive numbers indicate an average increase. All statistical analyses were performed in Stata/MP version 17.0 using the *itsa* package for ITS analysis.

## 3 Results

### 3.1 Number of trainees per month during the pre- and post-interruption periods

From April 2019 to March 2021, CHRS-affiliated ORPs held 6,325 naloxone training events. At those events, 101,332 people were trained and trainees were supplied with 295,067 doses of naloxone for use or community distribution. Among the trainees, 54.1% were lay responders, 21.5% were occupational responders, and 23.4% had unknown responder status. The number of trainees per month is summarized in the Supplemental Table.

There were 57,811 trainees during the pre-interruption period (Table 1). Approximately one-half were lay responders (50.8%) and 28% were occupational responders. The mean number of trainees per month during the pre-interruption period was 4,818, and the mean was higher among lay versus occupational responders. There were 43,521 trainees during the 12-month post-interruption period. Most (58.6%) were lay responders (*n* = 25,501) and 12.9% were occupational responders (*n* = 5,623).

**Table 1:** Number of people trained in overdose response and naloxone distribution before and after COVID-19 related stay-at-home orders in Maryland.

	N	Mean, per Month	Median	(Range)	% Change: Pre- vs. Post-Interruption
Pre-Interruption (Apr. 2019-Mar. 2020)					
Lay	29,343	2,445.25	2,211	(1,672–4,100)	—
Occupational	16,194	1,349.50	1,472	(623–1,729)	—
Unknown	12,274	1,022.83	741	(351–3,199)	—
Total	57,811	4,817.58	4,561	(3,203–7,259)	—
1-Month Post-Interruption (Apr. 2020-May 2020)					
Lay	2,293	191.08	(—)	(776–1,517)	−92.2%
Occupational	529	44.08	(—)	(234–295)	−96.7%
Unknown	1,092	91.00	(—)	(474–618)	−91.1%
Total	3,914	326.17	(—)	(14,84–2,430)	−93.2%
12-Months Post-Interruption (Apr. 2020-Mar. 2021)					
Lay	25,501	2,125.08	2,035	(776–3,265)	−13.1%
Occupational	5,623	468.58	444	(181–929)	−65.3%
Unknown	12,397	1,033.08	769	(368–2,863)	+1.0%
Total	43,521	3,626.75	3,410	(1,484–6,553)	−24.7%

When comparing the 1-month post-interruption period to the pre-interruption period, there was a 93.2% decrease in the average number of monthly trainees. A percent decrease of greater than 90% was observed for both lay and occupational responders. When comparing the 12-month post-interruption period to the pre-interruption period, the percent decrease in average number of monthly trainees was 24.7%. The percent decrease was higher for occupational responders (65.3%) than for lay responders (13.1%).

### 3.2 Estimated trends in the number of trainees

**Fig. 1:**
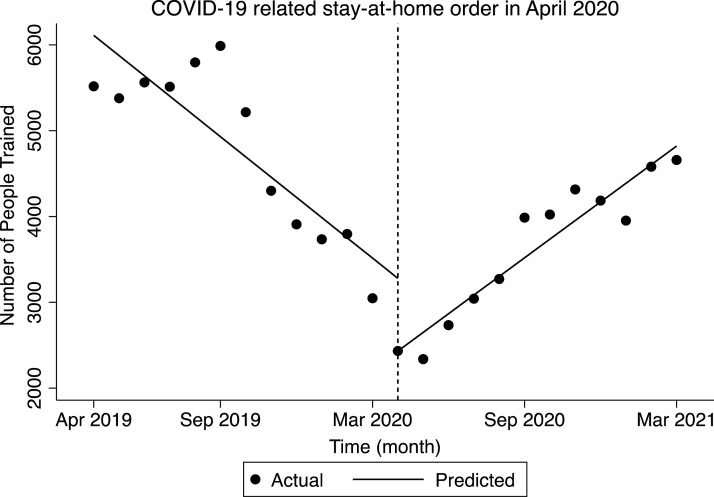
Plot of ITS model-based estimates of change in the average monthly number of trainees before and after COVID-19 related stay-at-home orders in Maryland.

**Fig. 2:**
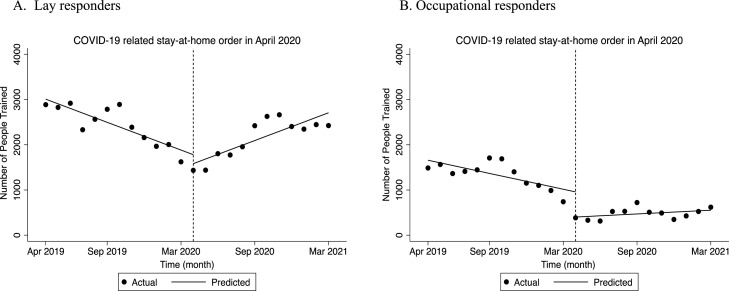
Plot of ITS model-based estimates of change in the average monthly number of trainees before and after COVID-19 related stay-at-home orders in Maryland, by type of responder.

Table 2 shows estimates of change in the average monthly number of trainees during the pre- and post-interruption periods that were derived from the ITS models; estimates are plotted in Fig. 1, Fig. 2. There was an estimated decrease in the monthly number of trainees during the pre-interruption period (−235, 95% CI: −329, −143) and the 1-month post-interruption period (−846, 95% CI: −1,490, −202). However, there was an estimated increase in the monthly number of trainees during the 12-month post-interruption period (+217, 95% CI: +176, +258).

**Table 2:** ITS model-based estimates of change in the average monthly number of trainees before and after COVID-19 related stay-at-home orders in Maryland.

	Coefficient	95% Confidence Interval	*p*-value
Pre-Interruption (Apr. 2019-Mar. 2020)			
Lay	−102	−132, −72	<0.001
Occupational	−58	−94, −23	0.003
Total	−235	−329, −143	<0.001
1-Month Post-Interruption (Apr. 2020-May 2020)			
Lay	−198	−551, +155	0.260
Occupational	−557	−878, −236	0.002
Total	−846	−1,490, −202	0.013
12-Months Post-Interruption (Apr. 2020-Mar. 2021)			
Lay	+102	+57, +147	<0.001
Occupational	+14	−3, +30	0.100
Total	+217	+176, +258	<0.001

Estimated decreases in the monthly number of trainees during the pre-interruption period were modest for both lay (−102, 95% CI: −132, −72) and occupational (−58, 95% CI: −94, −23) responders. By contrast, estimates of decreases during the 1-month post-interruption period were substantial among occupational responders (−557, 95% CI: −878, −236), but not among lay responders (−198, 95% CI: −551, +155). During the 12-month post-interruption period, estimates indicate an increase in the monthly number of trainees among lay responders (+102, 95% CI: +57, +147), but not among occupational responders (+14, 95% CI: −3, +30).

## 4 Discussion

Community-based programs that provide training in naloxone administration and give trainees naloxone to use and/or distribute to others are critical for overdose prevention. There are indications that there were disruptions to naloxone training programs during the emergence of the COVID-19 pandemic, and we explored trends in the number of trainees in the present study. Specifically, we investigated changes in average monthly number of people trained in overdose prevention before, during, and after the implementation of COVID-related stay-at-home orders in Maryland. Nearly one-half of the trainees were lay responders, 21.5% were occupational responders, and the rest had unknown responder status. We found a significant decrease in the number of people trained immediately following the pandemic-related stay-at-home orders, which appeared to be driven by a reduction in the number of occupational responders trained. There was a subsequent recovery in the number of trainees by March 2021, likely driven by increases in trainees who were lay responders. Our findings align with previous investigations that describe the impact of the COVID-19 pandemic on harm reduction service operations; specifically, several studies note reduced capacity to deliver naloxone training programs and missed patient contacts during suspension of services (McDonald et al., 2022; Noyes et al., 2021; Swann et al., 2022).

Training of both lay and occupational responders are an important component of maintaining naloxone availability in the community. We examined changes in training patterns separately among each group. During the study period, Maryland ORPs trained 54,844 lay responders and 21,817 occupational responders. There are several possible reasons why naloxone trainings declined among occupational responders but not lay responders. Occupational responders may have been forced to prioritize COVID-19 response activities, leaving less time for overdose prevention training. For example, law enforcement departments have reported suspension of in-service trainings, community outreach initiatives, and reassignment of personnel to high traffic public areas to maintain public order (Jennings and Perez, 2020). Reduced operation hours and pandemic-related service disruptions at harm reduction organizations including syringe service programs may have also affected trainings among some occupational responders during the pandemic. It is also plausible that hiring freezes entailed less training need for new employees of these organizations.

Trends in naloxone trainings among lay responders recovered quickly while trainings among occupational responders did not recover to pre-pandemic levels after 12 months. Lay responders trained through Maryland ORPs are reached primarily through harm reduction organizations such as syringe services programs and peer outreach organizations. Our findings underscore that harm reduction organizations may be more resilient and effective in reaching populations that are in need of naloxone especially in times of crisis. The rapid recovery of lay responder trainings within six months of the pandemic suggests that ORPs were able to adapt their operations. For example, some programs have added telephone-based, mail-based, and virtual naloxone trainings to their programming (Hughes et al., 2022; Krawczyk et al., 2021).

There was also a significant downtrend in naloxone trainings in the 12 months before implementation of the COVID-related stay-at-home order. A statewide expansion of the Naloxone Standing Order in 2017 eliminated naloxone training and prescription requirements for obtaining naloxone and expanded access to naloxone (Taylor et al., 2022). Although the standing order only allowed physicians and pharmacists to dispense naloxone without requiring a prescription or training, it may have inadvertently reduced demand for community-based naloxone trainings. It is also plausible that the demand for trainings stabilized by 2020 since community members and professionals may have received training in previous years. However, more research is needed to understand the effect of standing order policies on community naloxone training and distribution.

Our study has some limitations. Naloxone training data were provided by ORPs and delays in reporting could have led to measurement error. Data missingness due to lack of timely reporting could also have affected the quality of the data. To mitigate data quality issues, CHRS contacted program administrators at ORPs across the state to verify data. The dataset did not contain information on whether an individual receiving naloxone training was a first-time trainee and did not allow differentiation between first-time and repeat trainees. Number of people trained does not equate to the number of people reached because secondary naloxone distribution was common during the pandemic and may have led to an underestimate of the people reached. Generalizability of findings beyond Maryland may be limited. Finally, results should be viewed considering varied timing of stay-at-home orders versus broader closures in businesses, schools, and prohibitions on large social gatherings. While the stay-at-home order in Maryland ended in May 2020, some counties chose to extend stay-at-home orders while broader closures or limitations on operations largely continued across the state. The overall results indicate a decrease in naloxone trainings immediately after the implementation of the stay-at-home order, but the slower rebound among occupational responders may reflect continued limitations on some operations affecting occupational responders that may not have affected lay responders in the same way.

Nonetheless, this study examines a unique database and provides important public health implications. The decrease in naloxone trainings and slow recovery among occupational responders compared to lay responders indicate the need for strengthening the relationship between lay and occupational responders during public health crises. Lay responders have demonstrated their willingness to maintain naloxone supply in the community and play a crucial role in filling gaps in coverage. Therefore, it could be useful to explore models to increase engagement among lay individuals, such as community-based organizations or non-profits.

In case of future pandemics or other emergencies that occur while the opioid overdose epidemic continues, when traditional models of care may be inadmissible or inaccessible, local harm reduction organizations and health departments should consider targeted outreach services, online trainings, and mobile delivery of naloxone that proved successful at continuing access to naloxone (Bolinski et al., 2022). Programs should create contingency plans for staff and supply shortages by maintaining additional stock of naloxone and cross training staff members (Noyes et al., 2021). At the policy level, given that naloxone and other harm reduction services were designated as “essential services” during the pandemic, such organizations should be provided state and federal support and funding to continue operations. It is imperative to identify and fund strategies that were successful in supporting harm reduction organizations and broadening naloxone access to equip for future emergencies.

## 5 Conclusions

In this study conducted in Maryland, we found a decrease in community-based naloxone training immediately following the implementation of COVID-19 related stay-at-home order, driven by a substantial decrease in training among occupational responders. However, naloxone trainings recovered to pre-pandemic levels particularly among lay responders, indicating that overdose response programs are resilient and effectively adapted their operations to maintain naloxone access in the community.

## Declaration of Competing Interest

None

## Appendix Supplementary materials

Image, application 1
