# Supplementary material for: Disruptions to naloxone training among lay and occupational responders in Maryland during the emergence of COVID-19: Early impacts, recovery, and lessons learned
Source: Drug Alcohol Depend Rep. 2023 Jun 16;8:100173. doi: 10.1016/j.dadr.2023.100173 (PMC10271935; doi:10.1016/j.dadr.2023.100173)
Supplement: Supplementary file 1 [file mmc1.docx]

**Supplemental Table.** Number of people trained in overdose response and naloxone by month and responder status

| **Month & Year** | **Lay** | **Occupa-tional** | **Unknown** | **Total** |
| --- | --- | --- | --- | --- |
| Apr-19 | 1,672 | 1,542 | 562 | 3,776 |
| May-19 | 4,100 | 1,430 | 1,729 | 7,259 |
| Jun-19 | 2,705 | 1,720 | 675 | 5,100 |
| Jul-19 | 1,948 | 940 | 1,439 | 4,327 |
| Aug-19 | 2,341 | 1,571 | 3,199 | 7,111 |
| Sep-19 | 3,401 | 1,822 | 726 | 5,949 |
| Oct-19 | 2,611 | 1,729 | 563 | 4,903 |
| Nov-19 | 2,661 | 1,514 | 620 | 4,795 |
| Dec-19 | 1,890 | 961 | 351 | 3,202 |
| Jan-20 | 1,924 | 980 | 826 | 3,730 |
| Feb-20 | 2,081 | 1,362 | 829 | 4,272 |
| Mar-20 | 2,009 | 623 | 755 | 3,387 |
| Apr-20 | 776 | 234 | 474 | 1,484 |
| May-20 | 1,517 | 295 | 618 | 2,430 |
| Jun-20 | 2,021 | 464 | 617 | 3,102 |
| Jul-20 | 1,869 | 181 | 620 | 2,670 |
| Aug-20 | 1,430 | 929 | 997 | 3,356 |
| Sep-20 | 2,566 | 475 | 749 | 3,790 |
| Oct-20 | 3,265 | 762 | 788 | 4,815 |
| Nov-20 | 2,049 | 286 | 1,129 | 3,464 |
| Dec-20 | 2,673 | 424 | 1,570 | 4,667 |
| Jan-21 | 2,487 | 334 | 1,604 | 4,425 |
| Feb-21 | 1,876 | 521 | 368 | 2,765 |
| Mar-21 | 2,972 | 718 | 2,863 | 6,553 |
| Total | 54,844 | 21,817 | 24,671 | 101,332 |
